# Dupuytren’s contractures associated with the BRAF inhibitor vemurafenib: a case report

**DOI:** 10.1186/s13256-015-0634-4

**Published:** 2015-07-08

**Authors:** Sze Wai Chan, Daniel Alberto Vorobiof

**Affiliations:** Sandton Oncology Centre, 159 Rivonia Road, Morningside, Sandton, Johannesburg, Gauteng 2199 South Africa

**Keywords:** BRAF V600 mutation, Cutaneous malignant melanoma, Dupuytren’s contracture, Vemurafenib

## Abstract

**Introduction:**

Two previous cases of the development of Dupuytren’s contractures were reported in association with BRAF inhibitor treatment for BRAF V600E mutation-positive metastatic melanoma and metastatic papillary thyroid carcinoma. We reported on a third case with a slower onset of presentation.

**Case presentation:**

A 66-year-old white man was diagnosed with a BRAF V600E mutated metastatic cutaneous melanoma. He was commenced on oral vemurafenib 960mg twice daily. A marked response was achieved for his metastatic disease. He noticed a change of his hair characteristics and a feeling of “lumps” in both palms by 6 months. By 9 months, classical Dupuytren’s contracture was apparent.

**Conclusions:**

Dupuytren’s contracture is not a known side effect of BRAF inhibitor treatment. The timeline for the development of Dupuytren’s contracture on BRAF inhibitor treatment is not well defined. Although the etiology of Dupuytren’s contracture is unknown, an increase in tumor necrosis factor has been demonstrated to be a possible mechanism. BRAF inhibition has been shown to increase immune reaction in the tumor microenvironment and is associated with high serum tumor necrosis factor level. We propose that an increased level of tumor necrosis factor associated with BRAF inhibition may increase the risk of the development of Dupuytren’s contractures.

## Introduction

Vemurafenib, an oral anti-BRAF V600 kinase inhibitor, is indicated for the treatment of advanced malignant melanoma for patients whose tumors harbor the BRAF V600 mutation. Vemurafenib inhibits the MAP kinase pathway by binding to the kinase domain in mutant BRAF and has been shown to prolong both progression free and overall survival [1].

Toxicity from vemurafenib is common and includes many cutaneous side effects (skin rash, photosensitivity, hyperkeratosis, cutaneous squamous cell carcinoma, keratoacanthoma, and skin papilloma), alopecia, arthralgia, headache, fatigue, diarrhea and nausea [2–4].

Recently two cases of Dupuytren’s contractures have been reported in the medical literature in patients receiving a BRAF inhibitor [5, 6]. We report on an additional case, different in development when compared to the cases previously published.

## Case presentation

A 66-year-old white man was diagnosed with a BRAF V600E mutated metastatic cutaneous melanoma with subcutaneous metastases. He was known to have asthma for which he needed salbutamol and fluticasone inhalers. He had no other medical history of note. He was enrolled onto a national clinical trial and after signing an informed consent he was commenced on oral vemurafenib 960mg twice daily. A marked response was achieved (complete response) and his metastatic subcutaneous lesion disappeared after 5 months. He experienced grade 1 side effects such as arthralgia, a macular non-itchy skin rash over his upper chest, photosensitivity in sun exposed areas and general malaise. The appearance of hyperkeratotic lesions, keratoacanthomas and one basal cell carcinoma were treated with excisions, without a need to change his planned treatment dose.

**Fig. 1:**
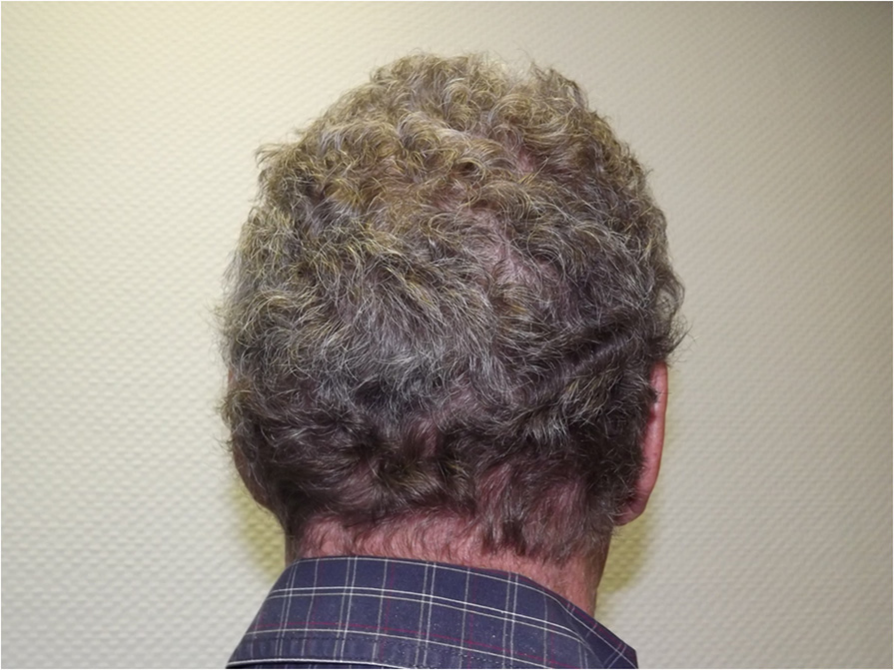
Development of curly hair on vemurafenib treatment

Approximately 6 months after the start of vemurafenib treatment, he noticed a change in his hair characteristics to curly hair (Fig. 1) and he started to feel “lumps” in both of his palms. By 9 months, most of his skin rash had disappeared and the “lumps” in his hands became noticeable and harder.

**Fig. 2:**
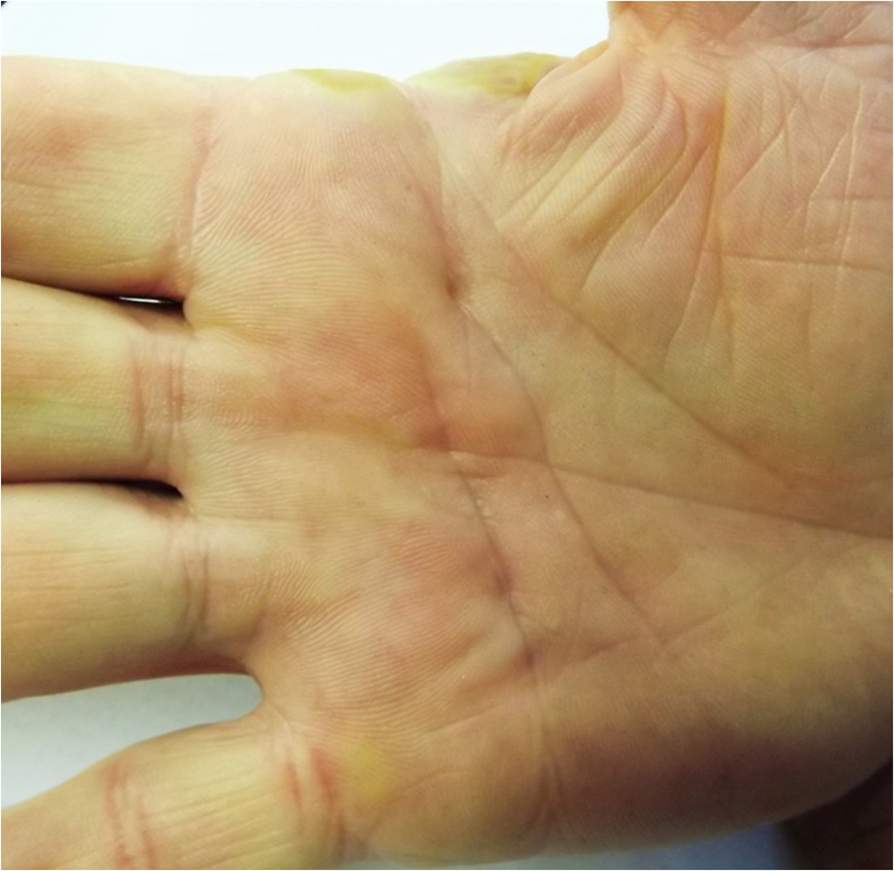
Development of fibrous band and palmar nodules suggestive of Dupuytren’s contractures

A clinical examination demonstrated painless nodules in both palms and formation of a fibrous band proximal to his 4th and 5th digits, consistent with a diagnosis of Dupuytren’s contractures (Fig. 2). There was no functional impairment with finger extension.

As he remained asymptomatic, a watch and wait approach was adopted with continuation of vemurafenib therapy.

## Discussion

Dupuytren’s contracture is a benign, slowly progressive fibrosis of the palmar fascia. It is a result of fibroblastic proliferation and disorderly collagen deposition. The early proliferative stage is associated with painless or painful nodules in the palms. With continued fibrosis, it will progress to form longitudinal bands or cords, limiting finger extension. Thumb and index fingers are usually spared and 4th and 5th fingers are commonly affected.

The etiology of Dupuytren’s contracture is unknown. Most patients present over the age of 50; it is more common in relatives of affected patients, male gender and people of European descent [7, 8]. There was no definitive association with a history of cigarette smoking, alcohol consumption or repetitive handling tasks. Our patient’s occupation was office-based and he enjoys gardening, fishing and golf.

On review of the current available medical literature, the first reported case of Dupuytren’s contractures secondary to BRAF kinase inhibitor therapy was reported by Bicknell *et al*. [5]. The described patient had a BRAF mutation-positive metastatic papillary thyroid carcinoma and was treated with a BRAF inhibitor on a clinical trial and developed Dupuytren’s contractures of both hands, palmar hyperkeratosis, a keratosis pilaris-like eruption and erythema nodosum [5].

Our patient presented with classical features of Dupuytren’s contractures such as palmar nodules and fibrous band formation, of a slow onset, manifesting at 6 months after commencement of vemurafenib treatment. He had neither flexion contractures nor functional impairment and his dosage of vemurafenib was not interrupted or discontinued.

This is also very different from a patient described by Sibaud and Chevreau where there was an abrupt onset of Dupuytren’s contractures after 6 weeks of vemurafenib treatment for metastatic melanoma with metastatic cervical lymph nodes [6]. The patient had a rapid development of fixed digital flexion contractures and visible cords but no associated palmar nodules. Discontinuation of vemurafenib was necessary due to the rapidly progressive nature and functional impairment affecting activities of daily living. There was however, no improvement or deterioration at 3 months after discontinuation of vemurafenib [6].

Dupuytren’s contracture is not a known side effect resulting from the BRAF kinase inhibitor vemurafenib. Sibaud and Chevreau hypothesized that vemurafenib induces a paradoxical proliferation in wild type-BRAF myofibroblasts [6]. BRAF inhibition has been shown to increase immune reaction in the tumor microenvironment. Wilmott *et al*. demonstrated that BRAF inhibitor-treated patients with metastatic melanoma have higher serum levels of interferon-γ (IFN-γ), CCL4, and tumor necrosis factor (TNF) [9]. Verjee *et al*. have shown that significant numbers of immune cells and a high level of pro-inflammatory cytokines are found in Dupuytren’s tissue where TNF selectively converted normal fibroblasts from the palms of patients with Dupuytren’s contractures into myofibroblasts via activation of the Wnt signaling pathway. Neutralizing antibodies to TNF reverse this phenomenon, suggesting that TNF inhibition may prevent progression or recurrence of Dupuytren’s contractures [10].

We propose that an increased level of TNF associated with BRAF inhibition may increase the risk of the development of Dupuytren’s contractures.

## Conclusions

The slow onset of the development of Dupuytren’s contracture in our patient suggested that the timeline for the development of Dupuytren’s contracture is not well defined on BRAF inhibitor treatment. An increased level of TNF associated with BRAF inhibition may increase the risk of the development of Dupuytren’s contractures. We need to be vigilant on this rare side effect throughout the course of the BRAF inhibitor treatment.

## Consent

Written informed consent was obtained from the patient for publication of this case report and any accompanying images. A copy of the written consent is available for review by the Editor-in-Chief of this journal.
